# A scoping Review of tools used to assess patient Complexity in rheumatic disease

**DOI:** 10.1111/hex.13200

**Published:** 2021-02-17

**Authors:** Kara Hawker, Cheryl Barnabe, Claire E.H. Barber

**Affiliations:** ^1^ Cumming School of Medicine University of Calgary Calgary AB Canada; ^2^ Arthritis Research Canada

**Keywords:** Care coordination, Rheumatoid Arthritis, Rheumatology, Systemic Lupus Erythematosus

## Abstract

**Objective:**

Patients with rheumatic diseases often have multiple comorbidities which may impact well‐being leading to high psychosocial complexity. This scoping review was undertaken to identify complexity measures/tools used in rheumatology that could help in planning and coordinating care.

**Methods:**

MEDLINE, EMBASE and CINAHL were searched from database inception to 14 December 2019 using keywords and Medical Subject Headings for “care coordination”, “complexity” and selected rheumatic diseases and known complexity measures/tools. Articles describing the development or use of complexity measures/tools in patients with adult rheumatologic diagnoses were included regardless of study design. Included articles were evaluated for risk of bias where applicable.

**Results:**

The search yielded 407 articles, 37 underwent full‐text review and 2 were identified during a hand search with 9 included articles. Only 2 complexity tools used in populations of adult patients with rheumatic disease were identified: the SLENQ and the INTERMED. The SLENQ is a 97‐item patient needs questionnaire developed for patients with systemic lupus (n = 1 study describing tool development) and applied in 5 cross‐sectional studies. Three studies (a practice article, trial and a cross‐sectional study) applied the INTERMED, a clinical interview to ascertain complexity and support coordinated care, in patients with rheumatologic diagnoses.

**Conclusions:**

There is limited information on the use of patient complexity measures/tools in rheumatology. Such tools could be applied to coordinate multidisciplinary care and improve patient experience and outcomes.

**Patient contribution:**

This scoping review will be presented to patient research partners involved in co‐designing a future study on patient complexity in rheumatic disease.

## 1 INTRODUCTION

Defining the burden of health issues on individuals and health systems is an evolving and nuanced field.^1^, ^2^ Since at least the 1970s, it has been proposed that biological, psychological and social factors influence prevention, causes, presentation, management and outcomes of diseases.^3^ This was conceptualized as a biopsychosocial model of medicine by Engel.^4^ Despite this long‐standing holistic model, for decades in medicine there has been an emphasis on measuring the burden of illness in terms of number and interrelatedness of various health conditions including measuring ‘comorbidities’ or ‘multimorbidity’,^1^, ^2^ with little attention paid to the interaction between health, illness and the social determinants of health in care delivery at an individual patient level.

An evolving concept is the notion of patient complexity.^2^ Patient complexity can be defined according to the vector model^5^ as the ‘interacting impact of biological, socioeconomic, cultural, environmental and behavioral forces as health determinants’. From a clinicians’ perspective, disease factors interact with psychosocial and environmental factors, creating challenges in clinical management, increasing time required in medical appointments and altering health‐care resource use.^2^ From a patient perspective, a model of cumulative complexity has been proposed by Shippee et al^6^ highlighting that patients experience a ‘workload’ of demands (job, social commitments, disease management) and have a ‘capacity’ (functional status, socio‐economic resources, literacy) to address these demands and that imbalance in workload vs demand contributes to complexity which can impact health outcomes.

Previous strategies to evaluate patient complexity have included examining the number of physician types involved in a patient's care and/or counting the number of a patient's comorbidities.^7^ Unfortunately, these strategies still lack integration of the psychosocial and environmental aspects of care needs, or an evaluation of the need for coordination of multiple services for individual patients. A match between one's case and care complexity is needed for efficient and effective patient‐centred health care and can be used to better direct patient support, care coordination and care plan monitoring.^8^

A variety of instruments have been developed to measure patient complexity across multiple health‐care contexts including ambulatory and acute care settings. These tools have been used in health‐care planning and care coordination, and can predict length of stay.^9^, ^10^ One of the earliest instruments was the INTERMED. Developed in the late 1990s, the INTERMED proposed a method for assessing past, present and future health service needs as a means of enhancing communication between health‐care professionals for patients with chronic disease to assist coordination of care and services.^11^ The information for completing the INTERMED is collected during a 15‐minute clinical interview as part of the medical history. It synthesizes data from biological, psychological, social and health‐care systems over time with each domain scored from ‘no vulnerability or need’ to ‘high vulnerability or need’ (Supplemental Table 1).^11^ Beyond its use in research, indicator colours, similar to those of a stop light, have been used to enhance communication to direct the need for health‐care team action items in different domains.^12^

Rheumatic diseases are typically systemic and therefore have the potential to impact multiple organ systems requiring lifelong frequent encounters with multiple health‐care providers. Complications of inflammatory rheumatic diseases are frequent and result in additional disease burden.^13^ People living with rheumatic diseases may experience challenges with mobility and physical functioning, which affect activities of daily living and can cause role limitation.^14^ Rheumatic disease can also cause significant personal and societal economic impacts as well as challenges with employment.^15^, ^16^, ^17^, ^18^ People living with rheumatic disease are also vulnerable to psychosocial challenges and concomitant mood disorders, including anxiety and depression. These common conditions contribute to decreased health‐related quality of life.^19^, ^20^, ^21^, ^22^ Socio‐economic status in turn may impact stress, depressive symptoms and disability as has been recently been evaluated in systemic lupus.^23^ Addressing a patient's psychosocial distress has been shown to improve coping and self‐efficacy, reduce psychological distress and reduce pain in arthritis patients.^24^ Patient complexity tools may help elucidate psychosocial challenges in a systematic fashion and can offer the care provider a holistic picture of the patient's experience beyond the physical manifestations of their disease and can be used to target interventions to improve health outcomes.^25^

The extent to which complexity tools have been used to better direct care coordination in rheumatology to improve health outcomes is unknown. The purpose of this scoping review was to understand and describe how patient complexity tools/measures have been used in rheumatic diseases to plan a future study aimed at improving care coordination and patient outcomes in rheumatology.

## 2 MATERIALS AND METHODS

**FIGURE 1:**
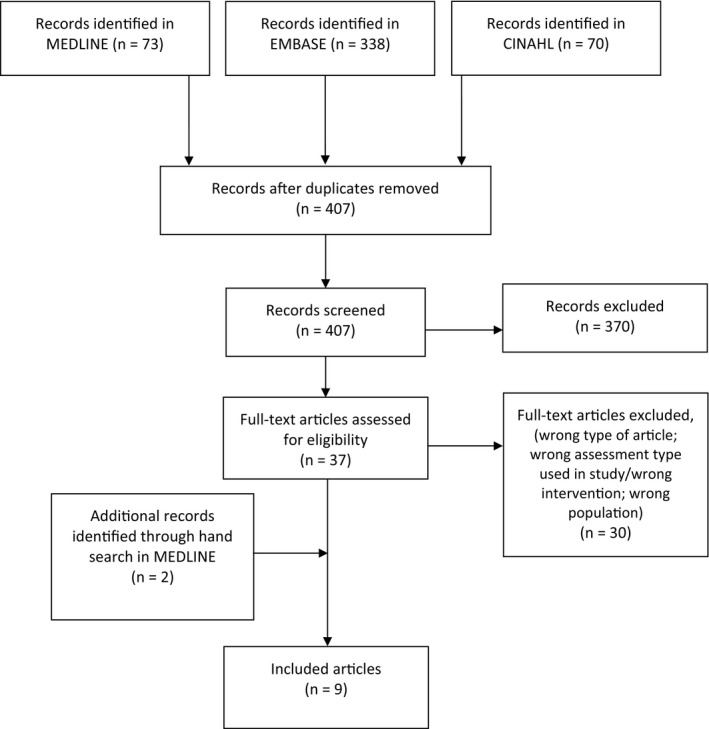
PRISMA flow diagram outlining iterative steps performed in this scoping review

A scoping review is an appropriate knowledge synthesis strategy for this research topic given the emerging nature of patient complexity as a construct, especially in rheumatic disease. Scoping reviews are used to provide a broad overview of evidence and this strategy is helpful in determining knowledge gaps to plan future research.^26^, ^27^ This scoping review was developed and reported according to the Preferred Reporting Items for Systematic Reviews and Meta‐Analyses extension for Scoping Reviews (PRISMA‐ScR).^28^, ^29^ A scoping review protocol was developed a priori (available on request). The search strategy was developed in consultation with a medical librarian following a series of iterative steps including 1) a preliminary search of MEDLINE and EMBASE to identify complexity tools and review text words and index terms for the identified articles; 2) a search using all identified keywords and search terms undertaken across three databases, MEDLINE, EMBASE and CINAHL, for articles from inception of the databases to 14 December 2019 and updated on 20 November 2020 (Figure 1 Supplementary material); and 3) the reference lists of identified articles were searched for any additionally relevant studies. Core search terms (MeSH and free text) were developed around two constructs: patient complexity, including existing complexity assessment tools, and survey terms. These were then combined with search terms specific to individual rheumatologic diseases in adults.

Title and abstract screening, as well as the article full‐text review, was conducted in duplicate independently by two authors (KH and CEHB) with any disagreements resolved by discussion. The following inclusion criteria were used: English language studies of any design were included if they developed, used or evaluated a tool to measure patient complexity in the context of an adult rheumatic disease including rheumatoid arthritis (RA), psoriatic arthritis, systemic lupus erythematosus (SLE) or vasculitis. Studies were excluded if they were non‐English, published in abstract form only or if they related to single constructs of patient care (eg disease activity, quality of life, functional status or medical comorbidity). Data were managed using Covidence (www.covidence.org). In addition, 15 authors of identified existing patient complexity tools found during our searches were contacted to identify any missed relevant publications in rheumatic disease.

Data extraction was performed independently in duplicate using a pilot‐tested data extraction form. Data pertaining to the article's identifying information, methods, population, interventions and outcomes were extracted.

Risk of bias of the included studies was assessed in duplicate using one of three tools depending on the study type. In the case of a disagreement in rating, consensus was reached through discussion. The Cochrane Risk of Bias tool^31^ was used to assess randomized controlled trials (RCTs). Domains of the tool were evaluated as ‘low’, ‘high’ or ‘unclear’ risk of bias. An overall risk of bias judgement was assigned.^31^ The COnsensus‐based Standards for the selection of health Measurement INstruments (COSMIN) Risk of Bias checklist^32^, ^33^, ^34^ was used to evaluate the methodologic quality of studies reporting on complexity measurement properties. Separate checklists for each relevant psychometric property were completed (eg content validity, structural validity, internal consistency, reliability and hypotheses testing), and the items were scored as ‘very good’, ‘adequate’, ‘doubtful’, ‘inadequate’ or ‘not applicable’, with the lowest item score for each property dictating the overall score.^32^, ^33^, ^34^ The overall rating was assigned using criteria for evaluation of the quality of results adapted from Prinsen et al^33^ as either ‘sufficient’, ‘insufficient’ or ‘indeterminate’. Quality of cross‐sectional and observational studies was assessed using the Quality Assessment Tool for Observational Cohort and Cross‐Sectional Studies from the National Heart, Lung, and Blood Institute (NHLBI).^35^ Items of the tool were evaluated ‘yes’, ‘no’, ‘not applicable’, ‘cannot determine’ or ‘not reported’, which helped guide provision of an overall rating for the quality of each study as ‘good’, ‘fair’ or ‘poor’.

## 3 RESULTS

A flow diagram of the search findings is presented in Figure 1. The search strategy returned 407 articles; 37 were identified as potentially relevant based on title and abstract screen, and 30 were excluded after full‐text review. A hand search identified two additional articles for a total of nine included articles. No additional articles were identified after contacting authors of existing complexity tools. Response rate from authors of existing tools was 60%.

Table 1 describes the characteristics of the nine included studies: one was in RA,^36^ one in general rheumatologic disease^25^ and seven in SLE.^12^, ^37^, ^38^, ^39^, ^40^, ^41^, ^42^ In SLE, one study described the development, and five described the application of the Systemic Lupus Erythematosus Needs Questionnaire (SLENQ) in SLE populations in New York, USA; New South Wales (NSW), Australia and the Netherlands.^37^, ^38^, ^39^, ^40^, ^41^, ^42^ The INTERMED was studied in three articles. The first was an observational study in RA where cluster analysis was completed based on patients’ INTERMED scores to determine clinical and disease associations with raw scores.^36^ An RCT used the INTERMED in a general rheumatology population to identify patients that were randomized to a tailored psychiatric intervention.^25^ One final article described the application of the INTERMED in a practice case with an SLE patient admitted to a gastroenterology ward to illustrate the utility of the tool but was not a research study.^12^

**TABLE 1:** Characteristics of included studies on use of patient complexity measures in rheumatic diseases

Author (Year)	Disease Population	Study Design	Setting	N	Location
*INTERMED*					
Koch et al (2001)^36^	RA	OS	Outpatient	75	Lausanne, Switzerland
Latour et al (2007)^12^	SLE	Practice article	Inpatient	1	Netherlands
Stiefel et al (2008)^25^	Rheumatic diseases and diabetes	RCT	Inpatients (rheum.) and outpatients (diabetes)	885	Lausanne, Switzerland
*SLENQ*					
Auerbach et al (2011)^37^	SLE	CS	Outpatient	378	New York, USA
Beckerman et al (2011)^38^	SLE	CS	Outpatient	378	New York, USA
Moses et al (2005)^39^	SLE	CS	Outpatient	386	NSW, Australia
Moses et al (2007)^40^	SLE	Tool development and testing	Outpatient	Varies^1^	NSW, Australia
Moses et al (2008)^41^	SLE	Repeated CS	Outpatient	386; 233^2^	NSW, Australia
Zirkzee et al (2014)^42^	SLE	CS	Outpatient	102	Netherlands

Abbreviation

Randomized controlled trial (RCT), cross‐sectional (CS), observational study (OS)

^1^ 84 SLE patients participated in focus groups and pre‐ and pilot testing phases in the development of the needs instrument; 386 SLE patients completed the SLENQ, and 47% (n = 144) of these patients completed the SLENQ a second time one week after the initial completion

^2^ 386 SLE patients completed the SLENQ survey at time 1, and 60% of those patients (n = 233) completed the same SLENQ survey at time 2 (6 months apart)

### 3.1 SLENQ: A tool to measure patient need in SLE

Moses et al^40^ describe the development of the SLENQ; the process included a literature review, interviews with health‐care professionals involved in caring for patients with SLE and focus groups involving SLE patients. The instrument includes 97 items along six domains: physical needs, daily living issues, social support, psychological needs, interpersonal communications, health information and access to services.^40^ Items are scored by the patient from 1 to 5 to indicate their need for support or care, where 1 represents no need and 5 represents high need. The instrument's face and content validity and acceptability were evaluated via an initial pre‐test and subsequent pilot study utilizing 20 and 44 SLE patients, respectively, registered within the Scleroderma/Lupus Resource Centre (patient support group). The SLENQ was well‐received by both convenience samples; all 97 items were retained in the final instrument. Construct and concurrent validity of the instrument was evaluated by SLE patients who completed both the SLENQ and the Medical Outcomes Study Short Form‐36 (MOS SF‐36).^43^, ^44^ Principal component analysis identified seven factors with eigenvalues > 1, accounting for 53% of total variance. Five of the seven SLENQ domains could be directly paired with the MOS SF‐36 domains, excluding general health, and these correlations were statistically significant. Only the health information and health services domains of the SLENQ had no equivalence with MOS SF‐36 domains. Test‐retest reliability was assessed utilizing 47% of the participants from the construct validity study who were willing to complete the SLENQ a second time. Reliability was confirmed as the criterion standard of internal consistency was exceeded (**α** > 0.7), and findings on 88% of items demonstrated moderate to substantial levels of agreement (κ > 0.4) and high level of test‐retest reliability.^40^

The remaining five articles utilizing the SLENQ were cross‐sectional survey studies. Two studies^37^, ^38^ highlighted patient characteristics associated with higher needs: patients with chronic symptoms or frequent flares, lower levels of education, those who were unemployed or receiving disability and those on social assistance. These studies also found that physical appearance changes (eg hair loss) and muscle pain were associated with higher patient‐reported needs. Moses et al^39^ reported that the most prevalent unmet needs were found in the physical domain with 84% of respondents reporting ‘moderate‐high’ needs. The top 10 highest levels of unmet needs were tiredness (81%), pain (73%), not being able to do things one used to do (72%), fear of exacerbation (72%), sleeping problems (70%), anxiety and stress (69%), feeling down (68%), fearing physical disability (65%), explaining SLE unpredictability to others (64%) and concerns regarding other health problems (63%).^39^ Zirkzee et al^42^ found that all patients responding to the survey reported a need in the physical domain. As well, a high total need score showed a trend for a low satisfaction with health‐care delivery; however, this was not statistically significant. Over time, through 2 repeated administrations of the SLENQ separated by a 6‐month window, Moses et al^41^ analysed the extent and variability of unmet care needs over time in SLE patients. For all participants, the top ten unmet needs were the same both times, with only slight changes in the order of most prevalent need. Similar to the previous study by Moses et al,^39^ tiredness followed by pain remained the top two unmet needs with a 6% and 2% reduction between times 1 and 2, respectively.^41^ The mean unmet need score declined significantly between time 1 (0.78) and time 2 (0.69). The mean unmet need score was significantly correlated with mean symptom score.^41^

### 3.2 Measuring patient complexity: the INTERMED

Koch et al^36^ is the only study that compared the utility of the INTERMED to conventional disease assessments in RA patients. A representative sample of RA patients from a tertiary care centre were evaluated using the INTERMED. Cluster analysis performed on their INTERMED scores formed two subgroups: complex and non‐complex. The complex cluster showed a trend for experiencing a longer duration of disease (*P* = .09), was more likely to be receiving disability compensation (*P* = .03) and scored worse on the MOS SF‐36 as demonstrated by the physical and mental component score (*P* < .01 and *P* = .04, respectively). These two clusters of patients also differed significantly on their subjective view of their health status, with the complex cluster rating themselves as more severely ill and having lower functional status. However, the clusters had similar scores on physician‐reported measures including their disease activity score (DAS 28), doctor's global assessment of disease, swollen joint count, tender joint count, blood sedimentation rate and radiographic score of hands and feet. The two clusters differed in health‐care utilization as well with the complex cluster experiencing higher frequencies of hospitalization (*P* = .04), more visits to the emergency room (*P* = .00), and more encounters with medical specialists (*P* = .01). Because the clusters did not differ in disease severity, the increased health‐care utilization demonstrated by the complex cluster was deemed to be likely due to case complexity and psychosocial vulnerabilities. The INTERMED appropriately identified complex patients with an elevated level of both disability and health‐care utilization. An INTERMED score of 21 has been proposed to identify patients who could benefit from case management.^45^ In this study, 29% of the patients scored at least 21, all of whom were in the complex cluster.^36^

Stiefel et al’s^25^ RCT used the INTERMED in patients admitted to the inpatient unit of the rheumatology service with inflammatory disease, degenerative disease, age‐related disease and other conditions, such as fibromyalgia. A cut‐off score of > 20 identified complex patients, and these patients were then randomized to the intervention or usual care (control) group. The intervention group involved up to three different interventions, single or combined, as proposed by a psychiatric nurse: supportive counselling from the psychiatric liaison nurse, referral to the liaison psychiatrist, and/or advice to the attending physician or organization of a multidisciplinary care conference. In the usual care group, the attending physician could still request a psychiatric consultation as part of routine management.

Of those screened (n = 701) with INTERMED, 35% (n = 247) qualified as complex and were included in the study (n = 125 randomized to intervention arm; n = 122 randomized to control arm). Overall, the intervention was associated with less depression [effect size in rheumatology patients: 1.7 (standard error (s.e.) = 1.1); *P* = .14] and higher levels of quality of life (QoL) [in both diabetic and rheumatology patients, 7.8 (s.e. = 1.6) higher score on QoL (F = 23.7; *P* < .001)] during follow‐up.^25^ Health‐care utilization did not differ between the two groups, with the exception of those that had to be hospitalized during follow‐up; this difference favoured the intervention, reaching significance at the 9‐month follow‐up assessment (*P* = .02).^25^

The third study which utilized the INTERMED was a practice case article by Latour et al^12^ describing the application of the INTERMED in a 27‐year‐old patient with a four‐year history of SLE. The article demonstrates the ability of the INTERMED to quantify, weigh and classify the complexity of a patient's problems. This article contrasts the INTERMED with the concept of a decision‐support system (DSS), which they define as a system ‘…designed to aid directly in clinical decision‐making, in which characteristics of the individual patients are used to generate patient‐specific assessments or recommendations that are then presented to clinicals for considerations’.^46^ Where DSSs focus on a single condition, the INTERMED assesses multiple health risks and needs via an interview that is based on the biopsychosocial model.^12^ The article illustrates how INTERMED can provide a quick, concise overview of a patient's health risks and needs, facilitates interdisciplinary communication and offers a framework for a treatment plan, describing the extent to which coordinated care is required.

### 3.3 Quality assessment of studies

There was a high risk of bias as measured by the Cochrane tool^31^ for the RCT by Stiefel et al^25^ (Supplemental Table 2). Concern for bias was present in the following domains: blinding for all outcomes; incomplete outcome data; and other possible sources of bias (including absent power calculation, potential selection bias).

The COSMIN checklist^32^, ^33^, ^34^ was used to evaluate the study by Moses et al^40^ for its measurement properties (Supplemental Tables 3, 4). Unfortunately, we were unable to access the full SLENQ, therefore our own rating of the questionnaire is unavailable, and the overall quality of the SLENQ was rated as indeterminate.

The NHLBI quality assessment tool^35^ was used to assess the methodological quality of the five cross‐sectional studies and one observational study^36^, ^37^, ^38^, ^39^, ^41^, ^42^ (Supplemental Table 5). Outcomes were used to assess the internal validity and risk of bias for each study, and the overall quality was rated. All six studies had an overall rating of fair.

## 4 DISCUSSION

This scoping review of complexity measures used in patients with rheumatic disease highlights a paucity of measures/tools used to evaluate patient complexity in this field and also emphasizes the evolving construct of patient complexity. Two tools were identified that have been investigated in populations with rheumatic disease to date: the INTERMED and the SLENQ.

As the SLENQ was developed specifically for patients with SLE, the study describing its development and psychometric properties was included in our review and evaluated using the COSMIN checklist. We have concluded that the tool was of indeterminate quality, limited by the inability to provide our own rating of the questionnaire as instructed by COSMIN. Nonetheless, the SLENQ is reported to be acceptable, valid and reliable.^40^ Advantages of the SLENQ include, tool development with affected patients, that it examines the range of needs in all life areas and that it is patient‐reported. Cross‐sectional studies of the SLENQ reveal high needs in many patients with SLE, with higher needs in those of lower socio‐economic status, lower education and in certain ethnic groups. Further study of the questionnaire may be warranted to determine the relationship to disease activity over time, other measures of complexity such as the INTERMED and how it can be used to direct patient care appropriately. The SLENQ is technically a measure of ‘patient need’; however, in the cumulative complexity model as defined by Shippee et al^6^ complexity from a patient perspective occurs from the interaction between ‘workload‐capacity demands’ imbalances. We posit that understanding patient needs along the social determinants of health may be one way of better understanding potential imbalances in ‘workload‐capacity demands’ and therefore understanding complexity from a patient‐centred perspective. Further work is clearly needed to better understand and define these concepts and their relationships in rheumatic disease.

As the INTERMED was not developed specifically for patients with rheumatic disease, the articles describing its development^11^ and psychometric properties, including its validity,^47^ predictive validity^48^, ^49^ and reliability,^11^ were not evaluated in the present scoping review. Nevertheless, based on the limited findings of our review it appeared to appropriately identify patients with rheumatic disease (primarily RA) who had higher patient complexity that was not explained by their disease activity and there was limited evidence that this tool could be used to direct psychosocial interventions to improve patient outcomes.

Through initial stages of our scoping review, while developing our search strategy, we identified a number of other complexity tools; however, none of these met the inclusion criteria as they had not been used in rheumatic disease populations. In general, these other tools are used to evaluate complexity, identify factors interfering with care, indicate multidisciplinary care and facilitate care coordination,^50^, ^51^, ^52^, ^53^, ^54^, ^55^, ^56^, ^57^ with the exception of three that were designed in particular for end‐of‐life care^58^, ^59^, ^60^ (Table 2). There is significant overlap between the domains of these other assessment tools with those of the INTERMED and SLENQ (Table 3). Of note, many of the tools described were derivations of the INTERMED and its adaptations including the Minnesota Complexity Assessment Method (MCAM)^52^ and the Patient Centered Assessment Method (PCAM).^56^

**TABLE 2:** Complexity tools and intended use

Complexity Tool	Full Name of Tool	Use of Tool
COMPRI^50^	Complexity Prediction Instrument	Indication for multidimensional assessment and interdisciplinary care coordination
FADOI‐Complimed^51^	Federation of Associations of Hospital Doctors on Internal Medicine‐Complimed	Evaluation of complexity
Hui's Criteria^58^	‐	Referral to palliative care specialist
IDC‐Pal^59^	Instrument in Diagnosing Complexity in Palliative Care	Determine level of palliative care provision
INTERMED^11^, ^47^	‐	Indication for multidisciplinary care
MCAM^52^, ^53^	Minnesota Complexity Assessment Method	Identify factors interfering with care; formulate new care plan
MECAM^54^	Minnesota Edinburgh Complexity Assessment Method	Identify factors posing risk to patient well‐being
OCCAM^55^	Oxford Case Complexity Assessment Method	Identify factors interfering with care; facilitate care coordination
PALCOM^60^	Predictive Model of Complexity in Early Palliative Care	Indication for specialized palliative care
PCAM^56^, ^57^	Patient Centered Assessment Method	Identify biopsychosocial complexities; make appropriate referrals
SLENQ^40^	Systemic Lupus Erythematosus Needs Questionnaire	Identify level of unmet need of care

Note

A hyphen in the column ‘Full Name of Tool’ indicates that the full name is the same as the abbreviated form of tool name

**TABLE 3:** Domains^1^ of exiting complexity tools

Tool	Administration	No. of items	Domains								
			Bio./Phys.	Social/ SES	Psych./Emot.	Ethical	Health care	Health literacy	ADL/Funct.	Comorbidity	Support req.
COMPRI (50)	HCP	117			✓		✓				✓
FADOI‐Complimed (51)	Patient	34							✓	✓	
Hui's Criteria (58)	HCP	47	✓	✓	✓		✓				
IDC‐Pal (59)	HCP	36	✓	✓			✓				
INTERMED (11, 47)	HCP	20	✓	✓	✓		✓				
MCAM (52, 53)	HCP	10	✓	✓	✓		✓				
MECAM (54)	HCP	11	✓	✓			✓	✓			
OCCAM (55)	HCP	27	✓	✓			✓		✓		
PALCOM (60)	Patient	5	✓	✓		✓		✓			
PCAM (56, 57)	HCP	12	✓	✓			✓	✓			
SLENQ (40)	Patient	97	✓	✓	✓	✓			✓		

Abbreviation

Complexity Prediction Instrument (COMPRI), Federal Association of Hospital Doctors on Internal Medicine‐Complimed (FADOI‐Complimed), Instrument for Diagnosing Complexity in Palliative Care (IDC‐Pal), Minnesota Complexity Assessment Method (MCAM), Minnesota Edinburgh Complexity Assessment Method (MECAM), Oxford Case Complexity Assessment Method (OCCAM), Predictive Model of Complexity in Early Palliative Care (PALCOM), Patient Centered Assessment Method (PCAM) and Systemic Lupus Erythematosus Needs Questionnaire (SLENQ)

^1^ Domain categorization based on our review of common domain themes including biological (Bio.), physical (Phys.), social (Soc.), socio‐economic status (SES), psychological (Psych.), emotional (Emot.), activities of daily living (ADL), functionality (Funct.), required (req.) and health‐care provider (HCP)

The presence of multiple chronic conditions is a growing reality for many patients with rheumatic diseases.^13^ Previous reports demonstrate that coordinated care delivered in the context of a team whose members form a cohesive unit can improve patient health‐care outcomes.^61^ More specifically, care coordination is associated with reduced hospital stays, lower costs for inpatients, less use of inpatient services and increased patient satisfaction.^62^ In the absence of care coordination programmes, clinicians work in parallel, instead of collaboratively, leaving the patient at risk for disjointed and ineffective care.^63^ Utilizing a complexity tool can help identify areas of care and/or support that patients need, and serve to guide care coordination in an interdisciplinary fashion. Unfortunately, no implementation studies of either tool were found demonstrating the successful use of these tools for care coordination, and this would be an important area for future study.

While, to our knowledge, this is the first scoping review of complexity measures in rheumatic diseases, there are some limitations to discuss. Extensive preliminary searches were conducted; however, the final search strategy did not include the term ‘patient needs’ as this concept was identified after the final search was completed. As such, it is possible articles using this term alone could have been missed in our review. While the SLENQ is described by the authors as a ‘patient's needs’ questionnaire and not as a complexity tool, it was included as the authors highlight that this tool can be used to aid in directing care to better meet patient needs and the domains overlapped significantly with other complexity tools identified. Further study is warranted to evaluate the correlation between the SLENQ and other established measures of complexity, which often focus on the clinicians’ perspectives of the psychosocial elements leading to higher patient complexity. Additionally, although we recognize a potential relationship between frailty and patient complexity, a search for frailty specific measures was beyond the scope of the present review. Similarly, we acknowledge that different types of complexity tools may be used in children and adolescents with rheumatic diseases, but this population was not included in the scope of our review. This review may also have missed non‐English language complexity tools and tools in clinical use that have not been published. We did not include the PsychInfo database in our search strategy given the focus on rheumatic disease and limited findings during test searches. Other research teams could consider including this database in future search strategies of patient complexity measures in more general patient populations. While the results of our work will be presented to patient research partners to plan future work in designing interventions to better coordinate rheumatology care, patient partners were not directly involved in this work and the framing of this work may therefore have been impacted. Finally, some of the quality assessment tools used to assess the studies were developed recently (eg COSMIN), which could have led to lower study ratings given emerging new standards for patient‐reported measure development and study reporting.

This review has identified two tools, the SLENQ and the INTERMED, which have been developed and/or tested in rheumatology patients. Though the SLENQ does not explicitly formulate a complexity score, it summarizes the magnitude of patient needs and the extent to which they are being met. The INTERMED can be used to direct patient care along various domains and also can produce a score that can be used to define complex versus non‐complex for care planning. However, the clinical utility and uptake of such tools are unknown and further study in this area is warranted. Additionally, potential avenues of future study include investigating the relationship between complexity measures and individual disease activity, functional status measures, and anxiety and depression screening scales to determine the set of measures that is most clinically useful in helping direct patient‐centred care while minimizing patient and clinician survey/interview burden. Evaluation of the utility of complexity tools such as the MCAM and PCAM used in other populations may be warranted in rheumatology patients. Lastly, future studies should evaluate the impact using complexity measures to direct care delivery on patient outcomes in rheumatology including patient experience with care, disease activity, patient activation, adherence and quality of life.

## CONFLICT OF INTEREST

The authors declare no conflicts of interest.

## Supporting information

Supplementary Material

## Data Availability

Data are available upon reasonable request to the authors.

## References

[1] Manning E , Gagnon M . The complex patient: a concept clarification. Nurs Health Sci. 2017;19(1):13‐21. 28054430 10.1111/nhs.12320

[2] Valderas JM , Starfield B , Sibbald B , Salisbury C , Roland M . Defining comorbidity: implications for understanding health and health services. Ann Fam Med. 2009;7(4):357‐363. 19597174 10.1370/afm.983 PMC2713155

[3] Engel GL . The need for a new medical model: a challenge for biomedicine. Science. 1977;196(4286):129‐136. 847460 10.1126/science.847460

[4] Engel GL . The clinical application of the biopsychosocial model. Am J Psychiatry. 1980;137(5):535‐544. 7369396 10.1176/ajp.137.5.535

[5] Safford MM , Allison JJ , Kiefe CI . Patient complexity: more than comorbidity. the vector model of complexity. J Gen Intern Med. 2007;22(Suppl 3):382‐390. 10.1007/s11606-007-0307-0 PMC2219701 18026806

[6] Shippee ND , Shah ND , May CR , Mair FS , Montori VM . Cumulative complexity: a functional, patient‐centered model of patient complexity can improve research and practice. J Clin Epidemiol. 2012;65(10):1041‐1051. 22910536 10.1016/j.jclinepi.2012.05.005

[7] Tonelli M , Wiebe N , Manns BJ , et al. Comparison of the complexity of patients seen by different medical subspecialists in a universal health care system. JAMA network open. 2018;1(7):e184852. 30646392 10.1001/jamanetworkopen.2018.4852 PMC6324421

[8] Miller AM , Swartwout KD , Schoeny ME , Vail M , McClenton R . Care coordination to target patient complexity and reduce disparities in primary care. Public Health Nurs. 2019;36(4):451‐460. 30895684 10.1111/phn.12606

[9] Kaiser KL , Hays BJ , Cho W‐J , Agrawal S . Examining health problems and intensity of need for care in family‐focused community and public health nursing. J Community Health Nurs. 2002;19(1):17‐32. 11985209 10.1207/S15327655JCHN1901_03

[10] de Jonge P , Huyse FJ , Stiefel FC , Slaets JPJ , Gans ROB . INTERMED—A clinical instrument for biopsychosocial assessment. Psychosomatics. 2001;42(2):106‐109. 11239122 10.1176/appi.psy.42.2.106

[11] Huyse FJ , Lyons JS , Stiefel FC , et al. “INTERMED”: a method to assess health service needs: I. Development and reliability. Gen Hosp Psychiatry. 1999;21(1):39‐48. 10068919 10.1016/s0163-8343(98)00057-7

[12] Latour CH , Huyse FJ , de Vos R , Stalman WA . A method to provide integrated care for complex medically ill patients: the INTERMED. Nurs Health Sci. 2007;9(2):150‐157. 17470190 10.1111/j.1442-2018.2007.00292.x

[13] Radner H , Yoshida K , Smolen JS , Solomon DH . Multimorbidity and rheumatic conditions—enhancing the concept of comorbidity. Nat Rev Rheumatol. 2014;10(4):252‐256. 24418765 10.1038/nrrheum.2013.212

[14] Salaffi F , Di Carlo M , Carotti M , Farah S , Ciapetti A , Gutierrez M . The impact of different rheumatic diseases on health‐related quality of life: a comparison with a selected sample of healthy individuals using SF‐36 questionnaire, EQ‐5D and SF‐6D utility values. Acta Biomed. 2019;89(4):541‐557. 30657123 10.23750/abm.v89i4.7298 PMC6502108

[15] Fazal SA , Khan M , Nishi SE , et al. A clinical update and global economic burden of rheumatoid arthritis. Endocr Metab Immune Disord Drug Targets. 2018;18(2):98‐109. 29141572 10.2174/1871530317666171114122417

[16] Li N , Chan E , Peterson S . The economic burden of depression among adults with rheumatoid arthritis in the United States. J Med Econ. 2019;22(4):372‐378. 30663460 10.1080/13696998.2019.1572015

[17] Carter EE , Barr SG , Clarke AE . The global burden of SLE: prevalence, health disparities and socioeconomic impact. Nat Rev Rheumatol. 2016;12(10):605‐620. 27558659 10.1038/nrrheum.2016.137

[18] Barra L , Borchin RL , Burroughs C , et al. Impact of vasculitis on employment and income. Clin Exp Rheumatol. 2018;36 Suppl 111(2);58‐64. PMC6003628 29352849

[19] Danoff‐Burg S , Friedberg F . Unmet needs of patients with systemic lupus erythematosus. Behav Med. 2009;35(1):5‐13. 19297299 10.3200/BMED.35.1.5-13 PMC2687813

[20] Dobkin PL , Fortin PR , Joseph L , Esdaile JM , Danoff DS , Clarke AE . Psychosocial contributors to mental and physical health in patients with systemic lupus erythematosus. Arthritis Rheum. 1998;11(1):23‐31. 10.1002/art.1790110105 9534490

[21] Philip EJ , Lindner H , Lederman L . Relationship of illness perceptions with depression among individuals diagnosed with lupus. Depress Anxiety. 2009;26(6):575‐582. 19242982 10.1002/da.20451

[22] Seawell AH , Danoff‐Burg S . Psychosocial research on systemic lupus erythematosus: a literature review. Lupus. 2004;13(12):891‐899. 15645742 10.1191/0961203304lu1083rr

[23] Sumner LA , Olmstead R , Azizoddin DR , et al. The contributions of socioeconomic status, perceived stress, and depression to disability in adults with systemic lupus erythematosus. Disabil Rehabil. 2020;42(9):1264‐1269. 30776317 10.1080/09638288.2018.1522550

[24] Backman C . Psychosocial aspects in the management of arthritis pain. Arthritis Res Ther. 2006;8(6). 10.1186/ar2083 PMC1794518 17169138

[25] Stiefel F , Zdrojewski C , Bel Hadj F , et al. Effects of a multifaceted psychiatric intervention targeted for the complex medically ill: a randomized controlled trial. Psychother Psychosom. 2008;77(4):247‐256. 18443391 10.1159/000129658

[26] Tricco AC , Lillie E , Zarin W , et al. A scoping review on the conduct and reporting of scoping reviews. BMC Med Res Methodol. 2016;16:15. 26857112 10.1186/s12874-016-0116-4 PMC4746911

[27] Grimshaw J , Canadian Institute for Health Research (CIHR) . A guide to knowledge synthesis‐ a knowledge synthesis chapter 2010 [cited 2020 November 20]. Available from: https://cihr‐irsc.gc.ca/e/41382.html

[28] Tricco AC , Lillie E , Zarin W , et al. PRISMA extension for scoping reviews (PRISMA‐ScR): checklist and explanation. Ann Intern Med. 2018;169(7):467‐473. 30178033 10.7326/M18-0850

[29] Peters MDJ , Godfrey CM , McInerney P , Munn Z , Tricco AC , Khalil H .Chapter 11: Scoping Reviews (2020 Version). In: Joanna Briggs Institute Reviewer’s Manual [Internet]. 2020. Available from: https://reviewersmanual.joannabriggs.org/

[30] Sterne JAC , Savović J , Page MJ , Elbers RG , Blencowe NS , Boutron I , et al.RoB 2: a revised tool for assessing risk of bias in randomised trials. 2019. 10.1136/bmj.l4898 31462531

[31] Higgins J , Savović J , Page MJ , Elbers RG , Sterne JAC .Chapter 8: Assessing risk of bias in a randomized trial. 2019. July 2019. In: Cochrane Handbook for Systematic Reviews of Interventions [Internet]. Version 6.0. Available from: www.training.cochrane.org/handbook

[32] Mokkink LB , de Vet HCW , Prinsen CAC , et al. COSMIN risk of bias checklist for systematic reviews of patient‐reported outcome measures. Qual Life Res. 2018;27(5):1171‐1179. 29260445 10.1007/s11136-017-1765-4 PMC5891552

[33] Prinsen CAC , Mokkink LB , Bouter LM , et al. COSMIN guideline for systematic reviews of patient‐reported outcome measures. Qual Life Res. 2018;27(5):1147‐1157. 29435801 10.1007/s11136-018-1798-3 PMC5891568

[34] Terwee CB , Prinsen CAC , Chiarotto A , et al. COSMIN methodology for evaluating the content validity of patient‐reported outcome measures: a Delphi study. Qual Life Res. 2018;27(5):1159‐1170. 29550964 10.1007/s11136-018-1829-0 PMC5891557

[35] National Heart L, and Blood Institute (NHLBI) . Study Quality Assessment Tools. Quality Assessment Tool for Observational Cohort and Cross‐Sectional Studies.

[36] Koch N , Stiefel F , Jonge PD , et al. Identification of case complexity and increased health care utilization in patients with rheumatoid arthritis. Arthritis Rheum. 2001;45(3):216‐221. 11409660 10.1002/1529-0131(200106)45:3<216::AID-ART251>3.0.CO;2-F

[37] Auerbach C , Beckerman NL . What social workers in health care should know about lupus: a structural equation model. Health Soc Work. 2011;36(4):269‐278. 22308879 10.1093/hsw/36.4.269

[38] Beckerman NL , Auerbach C , Blanco I . Psychosocial dimensions of SLE: implications for the health care team. J Multidiscip Healthc. 2011;4:63. 21594059 10.2147/JMDH.S19303 PMC3093952

[39] Moses N , Wiggers J , Nicholas C , Cockburn J . Prevalence and correlates of perceived unmet needs of people with systemic lupus erythematosus. Patient Educ Couns. 2005;57(1):30‐38. 15797150 10.1016/j.pec.2004.03.015

[40] Moses N , Wiggers J , Nicholas C , Cockburn J . Development and psychometric analysis of the systemic lupus erythematosus needs questionnaire (SLENQ). Qual Life Res. 2007;16(3):461‐466. 17091357 10.1007/s11136-006-9137-5

[41] Moses N , Wiggers J , Nicholas C . Persistence of unmet need for care among people with systemic lupus erythematosus: a longitudinal study. Qual Life Res. 2008;17(6):867‐876. 18553154 10.1007/s11136-008-9361-2

[42] Zirkzee EJM , Steup‐Beekman GM , Schouffoer AA , et al. Health care in systemic lupus erythematosus (SLE): the patient's perspective. Clin Rheumatol. 2014;33(9):1279‐1287. 24744153 10.1007/s10067-014-2595-1

[43] Ware JE , Sherbourne CD . The MOS 36‐item short‐form health survey (SF‐36). I. Conceptual framework and item selection. Med Care. 1992;30(6):473‐483. 1593914

[44] McHorney AC , Johne AW , Anastasiae AR . The MOS 36‐item short‐form health survey (SF‐36): II. Psychometric and clinical tests of validity in measuring physical and mental health constructs. Med Care. 1993;31(3):247‐263. 8450681 10.1097/00005650-199303000-00006

[45] De Jonge JP , Bauer HMI , Huyse HMF , Latour HMC . Medical inpatients at risk of extended hospital stay and poor discharge health status: detection with COMPRI and INTERMED. Psychosom Med. 2003;65(4):534‐541. 12883102 10.1097/01.psy.0000077504.01963.1b

[46] Hunt DL , Haynes RB , Hanna SE , Smith K . Effects of computer‐based clinical decision support systems on physician performance and patient outcomes: a systematic review. JAMA. 1998;280(15):1339‐1346. 9794315 10.1001/jama.280.15.1339

[47] Stiefel FC , de Jonge P , Huyse FJ , et al. "INTERMED": a method to assess health service needs ‐ II. results on its validity and clinical use. Gen Hosp Psych. 1999;21(1):49‐56. 10.1016/s0163-8343(98)00061-9 10068920

[48] Stiefel FC , de Jonge P , Huyse FJ , et al. INTERMED‐An assessment and classification system for case complexity: results in patients with low back pain. Spine. 1999;24(4):378‐384. 10065523 10.1097/00007632-199902150-00017

[49] Mazzocato C , Stiefel F , de Jonge P , Levorato A , Ducret S , Huyse FJ . Comprehensive assessment of patients in palliative care: a descriptive study utilizing the INTERMED. J Pain Symptom Manage. 2000;19(2):83‐90. 10699535 10.1016/s0885-3924(99)00156-6

[50] Huyse F , de Jonge P , Slaets J , et al. COMPRI: an instrument to detect patients with complex care needs. J Psychosomat Res. 2000;48(3):303. 10.1176/appi.psy.42.3.222 11351110

[51] Bonizzoni E , Gussoni G , Agnelli G , et al. The complexity of patients hospitalized in internal medicine wards evaluated by FADOI‐COMPLIMED score(s). A hypothetical approach. PLoS One. 2018;13(4):e0195805. 29659593 10.1371/journal.pone.0195805 PMC5901927

[52] Mount JK , Massanari RM , Teachman J . Patient care complexity as perceived by primary care physicians. Fam Syst Health. 2015;33(2):137‐145. 25893538 10.1037/fsh0000122 PMC4461487

[53] Peek CJ , Baird MA , Coleman E . Primary care for patient complexity, not only disease. Fam Syst Health. 2009;27(4):287‐302. 20047353 10.1037/a0018048

[54] Maxwell M , Hibberd C , Pratt R , Cameron I , Mercer S .Development and Initial Validation of the Minnesota Edinburgh Complexity Assessment Method (MECAM) for use within the Keep Well Health Check. Edinburgh, Scotland: NHS Health Scotland. 2011 November 30, 2011. Report No.

[55] Troigros O , Béjot Y , Rodriguez PM , Shoaib F , Ellis H , Wade D . Measuring complexity in neurological rehabilitation: the Oxford Case Complexity Assessment Measure (OCCAM). Clinical Rehabilitation. 2014;28(5):499‐507. 24275452 10.1177/0269215513505300

[56] Pratt R , Hibberd C , Cameron IM , Maxwell M . The Patient Centered Assessment Method (PCAM): Integrating the social dimensions of health into primary care. J Comorb. 2015;5(1):110‐119. 29090159 10.15256/joc.2015.5.35 PMC5636039

[57] Yoshida S , Matsushima M , Wakabayashi H , et al. Validity and reliability of the patient centred assessment method for patient complexity and relationship with hospital length of stay: a prospective cohort study. BMJ open. 2017;7(5):e016175. 10.1136/bmjopen-2017-016175 PMC5623372 28490567

[58] Hui D , Mori M , Watanabe SM , et al. Referral criteria for outpatient specialty palliative cancer care: an international consensus. Lancet Oncol. 2016;17(12):e552‐e559. 27924753 10.1016/S1470-2045(16)30577-0

[59] Martin‐Rosello LM , Sanz‐Amores RM , Salvador‐Comino RM . Instruments to evaluate complexity in end‐of‐life care. Curr Opin Support Palliat Care. 2018;12(4):480‐488. 30320622 10.1097/SPC.0000000000000403

[60] Tuca A , Gómez‐Martínez M , Prat A . Predictive model of complexity in early palliative care: a cohort of advanced cancer patients (PALCOM study). Support Care Cancer. 2018;26(1):241‐249. 28780728 10.1007/s00520-017-3840-3

[61] Grumbach K , Bodenheimer T . Can health care teams improve primary care practice? JAMA. 2004;291(10):1246‐1251. 15010447 10.1001/jama.291.10.1246

[62] Berry LL , Rock BL , Smith Houskamp B , Brueggeman J , Tucker L . Care coordination for patients with complex health profiles in inpatient and outpatient settings. Mayo Clin Proc. 2013;88(2):184‐194. 23290738 10.1016/j.mayocp.2012.10.016

[63] Stille CJ , Jerant A , Bell D , Meltzer D , Elmore JG . Coordinating care across diseases, settings, and clinicians: a key role for the generalist in practice. Ann Intern Med. 2005;142(8):700‐708. 15838089 10.7326/0003-4819-142-8-200504190-00038

