# Supplementary material for: A scoping Review of tools used to assess patient Complexity in rheumatic disease
Source: Health Expect. 2021 Feb 17;24(2):556–65. doi: 10.1111/hex.13200 (PMC8077158; doi:10.1111/hex.13200)
Supplement: Supplementary file 1 — Supplementary Material [file HEX-24-556-s001.docx]

Supplementary Material

**Supplemental Figure 1.** Example of search strategy used in MEDLINE database on December 14, 2020

1. Exp Arthritis, Rheumatoid/
2. Rheumat* arthriti*.tw,kf
3. RA.tw,kf
4. Arthriti* deforman*.tw,kf
5. Arthros* deforman*.tw,kf
6. Chronic ADJ2 polyarthriti*.tw,kf
7. Rheumarthriti*.tw,kf
8. Rheumat* polyarthriti*.tw,kf
9. Chronic articular rheumatism*.tw,kf
10. Caplan* syndrome*.tw,kf
11. Felty* syndrome*.tw,kf
12. Rheumatoid nodul*.tw,kf
13. Sjogren* syndrome*.tw,kf
14. Sicca syndrome*.tw,kf
15. Still* diseas*.tw,kf
16. Ankylosing spondyl*.tw,kf
17. Exp Lupus Erythematosus, Systemic/
18. Lupus.tw,kf
19. Erythematod* visceralis.tw,kf
20. SLE.tw,kf
21. Libman sack* diseas*.tw,kf
22. Exp Vasculitis/
23. Vasculiti*.tw,kf
24. Angi?ti*.tw,kf
25. Aortiti*.tw,kf
26. Arteriti*.tw,kf
27. Arter* ADJ2 inflam*.tw,kf
28. Behcet*.tw,kf
29. Cogan* syndrome*.tw,kf
30. Malignant atrophic papulos*.tw,kf
31. Degos* diseas*.tw,kf
32. Degos* syndrome*.tw,kf
33. Henoch ADJ1 scho?nlein.tw,kf
34. Purpura*.tw,kf
35. Thromboangi?tis obliteran*.tw,kf
36. Buerger* diseas*.tw,kf
37. Capillariti*.tw,kf
38. Eosinophilic granulomato?s*.tw,kf
39. Allergic granulomato?s*.tw,kf
40. EGPA*.tw,kf
41. Churg-Strauss*.tw,kf
42. Granulomato?s* with polyangi?ti*.tw,kf
43. Wegener*.tw,kf
44. Polyarteritis nodos*.tw,kf
45. Periarteritis nodos*.tw,kf
46. Takayasu*.tw,kf
47. Microscopic polyangi?ti*.tw,kf
48. Vascular inflam*.tw,kf
49. Anti-glomerular basement membrane diseas*.tw,kf
50. Anti-GBM diseas*.tw,kf
51. Goodpasture* diseas*.tw,kf
52. Exp Arthritis, Psoriatic/
53. Psoria* ADJ1 arthriti*.tw,kf
54. Arthropathic psoria*.tw,kf
55. Psoria* polyarthriti*.tw,kf
56. Psoria* ADJ1 arthro*.tw,kf
57. Psoria* rheumat*.tw,kf
58. OR/1-57
59. Complexity.tw,kf
60. Utilization.tw,kf
61. Psycho?social.tw,kf
62. Patient* ADJ2 complex*.tw,kf
63. Clinical ADJ2 complex*.tw,kf
64. Case ADJ2 complex*.tw,kf
65. Care ADJ2 delivery.tw,kf
66. Care ADJ2 model*.tw,kf
67. Care ADJ2 coordinat*.tw,kf
68. Care ADJ2 plan*.tw,kf
69. Care ADJ2 organi*.tw,kf
70. Care ADJ2 harmoni*.tw,kf
71. Patient care ADJ2 team*.tw,kf
72. Comorbidity/
73. Multimorbidity/
74. Multiple chronic conditions/
75. Comorbidit*.tw,kf
76. Co-morbidit*.tw,kf
77. Multimorbidit*.tw,kf
78. Multi-morbidit*.tw,kf
79. Multi?disciplin* ADJ2 care.tw,kf
80. Inter?disciplin* ADJ2 care.tw,kf
81. Integrat* ADJ2 care.tw,kf
82. OR/59-81
83. Patient Reported Outcome Measures/
84. Needs assessment/
85. INTERMED.tw,kf
86. Patient cent?red assessment method.tw,kf
87. Minnesota complexity assessment method.tw,kf
88. Oxford case complexity assessment measure.tw,kf
89. Hui* criteri*.tw,kf
90. PALCOM.tw,kf
91. IDC-Pal.tw,kf
92. (Activation and coordination) adj team.tw,kf
93. Vector model*.tw,kf
94. Omaha system.tw,kf
95. FADOI-COMPLIMED.tw,kf
96. Cumulative illness rating scale*.tw,kf
97. Complex* ADJ2 model*.tw,kf
98. Complex* ADJ2 method*.tw,kf
99. Complex* ADJ2 framework*.tw,kf
100. Complex* ADJ2 assess*.tw,kf
101. Complex* ADJ2 tool*.tw,kf
102. Complex* ADJ2 scal*.tw,kf
103. OR/83-102
104. 58 and 82 and 103

**Supplemental Table 1.** Variables of the INTERMED schema utilized to produce INTERMED score

| **Domain** | **History** | **Current state** | **Prognosis** |
| --- | --- | --- | --- |
| Biological | Chronicity  Diagnostic dilemma | Severity of symptoms  Diagnostic challenge | Complications and life threat |
| Psychological | Restrictions in coping  Psychiatric dysfunction | Resistance to treatment  Psychiatric symptoms | Mental health threat |
| Social | Restrictions in integration  Social dysfunctioning | Residential instability  Restrictions of network | Social vulnerability |
| Health care | Intensity of prior treatment  Prior treatment experience | Organization of care  Appropriateness of referral | Coordination of health care |

Created with permission of Dr. Annette Boenink, contact for the INTERMED Consortium ([www.intermedconsortium.com](http://www.intermedconsortium.com))

**Supplemental Table 2.** Cochrane risk of bias comparison for RCT by Stiefel et al. (1)

| **Domain** | **Risk of Bias** | **Comments** | |
| --- | --- | --- | --- |
| Sequence generation | Low | | Computer-generated randomization |
| Allocation concealment | Low | | Participants and investigators enrolling participants unable to foresee assignment due to central allocation |
| Blinding of participants and personnel for All outcomes | High | | High risk of incomplete blinding due to study design |
| Blinding of outcome assessors for All outcomes | Low | | Follow-up performed by nurse blinded to intervention |
| Incomplete outcome data for All outcomes | Unclear | | Low risk would be assigned if either no missing data, reasons for missing data unlikely related to outcome, missing data inputted using acceptable method |
| Selective outcome reporting | Low | | Reporting results are consistent with methods |
| Other sources of bias | Unclear | | No power calculation reported; unclear if study adequately powered especially for subgroup analyses |

*From:* Higgins J, Savović J, Page MJ, Elbers RG, Sterne JAC. Chapter 8: Assessing risk of bias in a randomized trial. In: Higgins J, Thomas J, Chandler J, Cumpston M, Li T, Page MJ, et al., editors. Cochrane Handbook for Systematic Reviews of Interventions. Version 6.0 ed; 2019.

**Supplemental Table 3.** COSMIN criteria and rating system for evaluating content validity of the SLENQ

| **PROM:** Systemic Lupus Erythematosus Needs Questionnaire (SLENQ) | **PROM development study** | **Content validity study 1** | **Rating of reviewers** | **OVERALL RATING OF SLENQ** | **QUALITY OF EVIDENCE** |
| --- | --- | --- | --- | --- | --- |
| **Criteria*** | + / - / ± / ? | + / - / ± / ? | + / - / ± / ? | + / - / ± | High, moderate, low, very low |
| **Relevance** |  |  |  |  |  |
| 1.Are the included items relevant for the construct of interest? | - | - | NA |  |  |
| 2.Are the included items relevant for the target population of interest? | ? | - | NA |  |  |
| 3.Are the included items relevant for the context of use of interest? | + | - | NA |  |  |
| 4.Are the response options appropriate? | + | - | NA |  |  |
| 5.Is the recall period appropriate? | ? | - | NA |  |  |
| **RELEVANCE RATING** | ? | - | NA | ? | Very Low |
| **Comprehensiveness** |  |  |  |  |  |
| 6.Are all key concepts included? | - | - | NA |  |  |
| **COMPREHENSIVENESS RATING** | - | - | NA | ? | Very Low |
| **Comprehensibility** |  |  |  |  |  |
| 7.Are the PROM instructions understood by the population of interest as intended? | - | - |  |  |  |
| 8.Are the PROM items and response options understood by the population of interest as intended? | - | - |  |  |  |
| 9.Are the PROM items appropriately worded? |  |  | NA |  |  |
| 10.Do the response options match the question? |  |  | NA |  |  |
| **COMPREHENSIBILITY RATING** | - | - | NA | ? | Very Low |
| **CONTENT VALIDITY RATING** | ? | - | NA | ? | Low |

(+) = sufficient; (-) = insufficient; (±) = inconsistent; (?) = indeterminate; NA = not applicable

From: Terwee C, Prinsen C, Chiarotto A, Westerman M, Patrick D, Alonso J, et al. COSMIN methodology for evaluating the content validity of patient-reported outcome measures: a Delphi study. Qual Life Res. 2018;27(5):1159-70.

**Supplemental Table 4.** COSMIN risk of bias evaluation of SLENQ using developmental and psychometric property study of questionnaire by Moses et al. (2)

| **PROM** | **Box 3. Structural Validity** | | | **Box 4. Internal Consistency** | | | **Box 6. Reliability** | | | **Box 9. Hypotheses Testing for Construct Validity** | | |
| --- | --- | --- | --- | --- | --- | --- | --- | --- | --- | --- | --- | --- |
|  | n | Meth qual | Result (rating) | n | Meth qual | Result (rating) | n | Meth qual | Result (rating) | n | Meth qual | Result (rating) |
| SLENQ | 386 | Inadequate | 7 factors* with eigenvalue > 1 accounting for 53% of total variance (-) | 386 | Very good | α = 0.77-0.96 (?) | 165 | Doubtful | κ = 0.32-0.70 (?) | N/a | Adequate | Correlation of domains with SF-36 = -61 to -0.31 (?) |

(+) = sufficient; (-) = insufficient; (?) = indeterminate
*Seven factors include: Psychological/spiritual/existential; Health services; Health information; Physical; Social support; Daily living; Employment/financial

*From:* Mokkink LB, De Vet HCW, Prinsen CAC, Patrick DL, Alonso Caballero J, Bouter LM, et al. COSMIN Risk of Bias checklist for systematic reviews of Patient-Reported Outcome Measures. Qual Life Res. 2018;27(5):1171-9.

Prinsen C, Mokkink L, Bouter L, Alonso J, Patrick D, Vet H, et al. COSMIN guideline for systematic reviews of patient-reported outcome measures. Qual Life Res. 2018;27(5):1147-57.

| **Supplemental Table** **5**. NHLBI quality assessment for observational cohort and cross-sectional studies | | | | | | |
| --- | --- | --- | --- | --- | --- | --- |
| **Criteria** | **Auerbach et al.** (3) | **Beckerman et al.** (4) | **Koch et al.** (5) | **Moses et al.** (6) | **Moses et al.** (7) | **Zirkzee et al.** (8) |
| 1.Was the research question or objective in this paper clearly stated? | Y | Y | Y | Y | Y | Y |
| 2.Was the study population clearly specified and defined? | Y | Y | N | Y | Y | Y |
| 3.Was the participation rate of eligible persons at least 50% | N | N | CD | Y | Y | Y |
| 4.Were all the subjects selected or recruited from the same or similar populations (including same time period)? Were inclusion and exclusion criteria for being in the study pre-specified and applied uniformly to all participants? | Y | Y | CD | Y | Y | Y |
| 5.Was a sample size justification, power description, or variance and effect estimates provided? | NR | N | N | N | N | N |
| 6. For the analyses in this paper, were the exposure(s) of interest measured prior to the outcome(s) being measured? | N | N | N | N | N | N |
| 7. Was the time frame sufficient so that one could reasonably expect to see an association between exposure and outcome if it existed? | N | N | N | N | N | N |
| 8. For exposures that can vary in amount or level, did the study examine different levels of the exposure as related to outcome (eg, categories of exposure, or exposure measured as continuous variable)? | N | N | CD | N | N | Y |
| 9.Were the exposure measures (independent variables) clearly defined, valid, reliable, and implemented consistently across all study participants? | Y | Y | Y | Y | Y | Y |
| 10. Was the exposure(s) assessed more than once over time? | NA | NA | N | NA | N | NA |
| 11.Were the outcome measures (dependent variables) clearly defined, valid, reliable, and implemented consistently across all study participants? | Y | Y | Y | Y | Y | CD |
| 12.Were the outcome assessors blinded to exposure status of participants? | NA | NA | NA | NA | NA | NA |
| 13. Was loss to follow-up after baseline 20% or less? | NA | NA | Y | NA | N | NA |
| 14.Were key potential confounding variables measured and adjusted statistically for their impact on the relationship between exposure(s) and outcome(s)? | Y | Y | Y | Y | Y | Y |
| Overall rating | Fair | Fair | Fair | Fair | Fair | Fair |

Y = Yes; N = No; NA = Not applicable; NR = Not reported

*Available at:* https://www.nhlbi.nih.gov/health-topics/study-quality-assessment-tools

1. Stiefel F, Zdrojewski C, Bel Hadj F, Boffa D, Dorogi Y, So A, et al. Effects of a multifaceted psychiatric intervention targeted for the complex medically ill: a randomized controlled trial. Psychother Psychosom. 2008;77(4):247-56.

2. Moses N, Wiggers J, Nicholas C, Cockburn J. Development and psychometric analysis of the systemic lupus erythematosus needs questionnaire (SLENQ). Qual Life Res. 2007;16(3):461-6.

3. Auerbach C, Beckerman NL. What social workers in health care should know about lupus: a structural equation model. Health Soc Work. 2011;36(4):269-78.

4. Beckerman NL, Auerbach C, Blanco I. Psychosocial dimensions of SLE: implications for the health care team. Journal of multidisciplinary healthcare. 2011;4:63.

5. Koch N, Stiefel F, de Jonge P, Fransen J, Chamot AM, Gerster JC, et al. Identification of case complexity and increased health care utilization in patients with rheumatoid arthritis. Arthritis Rheum. 2001;45(3):216-21.

6. Moses N, Wiggers J, Nicholas C, Cockburn J. Prevalence and correlates of perceived unmet needs of people with systemic lupus erythematosus. Patient Educ Couns. 2005;57(1):30-8.

7. Moses N, Wiggers J, Nicholas C, Moses N, Wiggers J, Nicholas C. Persistence of unmet need for care among people with systemic lupus erythematosus: a longitudinal study. Qual Life Res. 2008;17(6):867-76.

8. Zirkzee EJ, Steup-Beekman GM, Schouffoer AA, Henquet SM, Caljouw MA, Huizinga TW, et al. Health care in systemic lupus erythematosus (SLE): the patient's perspective. Clin Rheumatol. 2014;33(9):1279-87.
